# Dysfunction of Synaptic Vesicle Endocytosis in Parkinson’s Disease

**DOI:** 10.3389/fnint.2021.619160

**Published:** 2021-05-20

**Authors:** Li Zou, Ye Tian, Zhentao Zhang

**Affiliations:** Department of Neurology, Renmin Hospital of Wuhan University, Wuhan, China

**Keywords:** Parkinson’s disease, synaptic dysfunction, neurodegeneration, α-synuclein, SYNJ1

## Abstract

Parkinson’s disease (PD) is the second most common neurodegenerative disorder after Alzheimer’s disease. It is a chronic and progressive disorder estimated to affect at least 4 million people worldwide. Although the etiology of PD remains unclear, it has been found that the dysfunction of synaptic vesicle endocytosis (SVE) in neural terminal happens before the loss of dopaminergic neurons. Recently, accumulating evidence reveals that the PD-linked synaptic genes, including *DNAJC6*, *SYNJ1*, and *SH3GL2*, significantly contribute to the disruptions of SVE, which is vital for the pathogenesis of PD. In addition, the proteins encoded by other PD-associated genes such as *SNCA*, *LRRK2*, *PRKN*, and *DJ-1* also play key roles in the regulation of SVE. Here we present the facts about SVE-related genes and discussed their potential relevance to the pathogenesis of PD.

## Introduction

Parkinson’s disease (PD) is the second most common neurodegenerative disease after Alzheimer’s disease (AD) (De Lau and Breteler, 2006). The prevalence of PD in those older than 65 is about 1.7%. PD is usually diagnosed based on motor symptoms (e.g., rest tremor, bradykinesia, rigidity, etc.). The pathological characteristics of PD are the degeneration and loss of dopaminergic neurons in the nigrostriatal pathway. However, the pathogenesis of PD remains unclear. Studies have confirmed that in PD, neuronal axon degeneration and/or synaptic dysfunction prior to the loss of dopamine neurons (Burke and O’malley, 2013; Kurowska et al., 2016). Most synaptic functions are effective by the release and reuptake of neurotransmitters stored in the synaptic vesicles. The recycling of synaptic vesicles plays a decisive role in the function of the synapse. Increasing evidence suggests that dysfunction of synapse may play an important role in the onset and progression of PD. Synaptic dysfunction happens in the early stage of PD and may be one of the causes to trigger the loss of dopaminergic neurons (Schirinzi et al., 2016). Several genes that regulate synaptic function are found to be involved in the pathological process of PD, highlighting the importance of further studies on synaptic dysfunction in PD. Therefore, we briefly discuss the experimental and genetic evidence on synaptic dysfunction in PD, in order to provide a prospective view on the treatment of PD.

## Synaptic Vesicle Recycling

**FIGURE 1:**
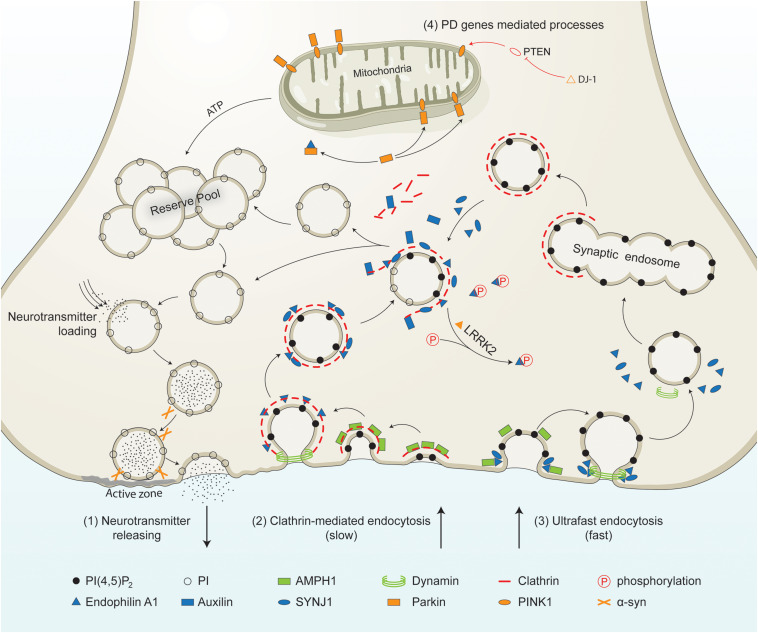
PD-linked and SVE-associated genes in synaptic vesicle recycling. Schematic representation of a presynaptic terminal showing the roles of PD-linked genes (orange) and SVE-associated genes (blue) in synaptic vesicle recycling. It shows the neurotransmitter release process, clathrin-mediated endocytosis, ultrafast endocytosis, and other processes in which PD genes are involved. (1) Neurotransmitter releasing. Synaptic vesicles from the reserve pool or derived from the uncoating process are loaded with neurotransmitters. The vesicles move toward the active zone and then fuse to the plasma membrane in the presence of synaptic proteins including α-syn. Neurotransmitters are released into the synaptic cleft. (2) Clathrin-mediated endocytosis. This process is initiated by the coating of the plasma membrane by clathrin. The coated membrane is curved via the activity of AMPH1. Then endophilin mediates the invagination of the clathrin-coated plasma membrane. Endophilin A1 recruits dynamin to the neck of the clathrin-coated vesicles (CCVs). Endophilin A1 contains several SH3 domains and interacts with SYNJ1 and/or Parkin. Once recruited, SYNJ1 dephosphorylates PI(4,5P)_2_ into PI, shedding clathrin and its adaptor from the bilayer. LRRK2 phosphorylates endophilin, leading to dissociation of the later from CCVs. The removal of the clathrin coat is mediated by auxilin. Finally, the uncoated endocytic vesicles move into the reserve pool, or directly undergo loading and releasing process. (3) Ultrafast endocytosis. During this type of endocytosis, AMPH1 and endophilin A1 are recruited to the plasma membrane, and regulate the curvature of the emerging vesicle. Dynamin then constricts the neck and mediates synaptic vesicle fission from the plasma membrane. The large endocytic vesicles cluster and fuse to form synaptic endosomes. The synaptic endosomes are further coated by clathrin to generate CCVs, which undergo clathrin-uncoating and generate endocytic vesicles. (4) Other processes mediated by PD-related genes. PTEN is a lipid phosphatase that is inhibited by DJ-1. PTEN increases the level of PINK1 in the mitochondrial. PINK1 is released by the mitochondria and triggers neuronal differentiation. The PTEN-Parkin axis is necessary for proper mitochondrial function. This guarantees ATP synthesis and is necessary for most processes, such as vesicle fusion and the mobilization of the vesicles in the reserve pool during synaptic vesicle recycling.PI(4,5)P_2_, phosphatidylinositol 4,5-bisphosphate; PI, phosphatidylinositol; α-syn, alpha-synuclein; AMPH1, amphiphysin 1; SYNJ1, synaptojanin 1; LRRK2, leucine-rich repeat serine/threonine protein kinase 2; PTEN, phosphatase and tensin homolog; PINK1, PTEN induced putative kinase 1; DJ-1, Parkinson’s disease protein 7; ATP, adenosine triphosphate.

Presynaptic terminals are highly differentiated structures in which synaptic vesicles (SVs) accumulate near the electron-dense active region and are physically and biochemically prepared for fusion and recycling under the action of a highly conserved group of molecules (Rizzoli and Betz, 2005; Hua et al., 2011; Imig et al., 2014). The activity of presynaptic vesicles can be approximately divided into the following main steps (Südhof, 2004; Watanabe et al., 2014; Watanabe and Boucrot, 2017): (1) Neurotransmitters are pumped into the vesicles through the neurotransmitter transporter on the surface of the vesicles; (2) The vesicles are transported to the near-activated area of the presynaptic neuron end, where they are accumulated relatively; (3) The vesicles to be released are spatially close to or directly anchored to the active zone of presynaptic plasma membrane; (4) The action potential is transmitted to the axis at the terminal end, the voltage-dependent calcium channel opening of the presynaptic membrane causes calcium influx; (5) High concentration of calcium ions fuse the vesicle with the presynaptic membrane and forms an opening, and the neurotransmitter is released through the opening. (6) Endocytosis of vesicles after fusion with the presynaptic membrane is thought to have two pathways: faster clathrin-independent endocytic processes, like ultrafast endocytosis, and slower clathrin-mediated endocytosis; (7) Neurotransmitters are re-introduced into the vesicles after endocytosis by the neurotransmitter transporter (see Figure 1).

There are also other modes for synaptic vesicle retrieval, including kiss-and-run and several variations of bulk endocytosis such as ultrafast and activity-dependent bulk endocytosis (Saheki and De Camilli, 2012; Soykan et al., 2016; Cousin, 2017; Milosevic, 2018; Chanaday et al., 2019). Recently, more studies consider that bulk retrieval pathways are also important rather than as an addition (Soykan et al., 2016; Chanaday et al., 2019). These modes of endocytosis have also been found to be regulated by proteins like dynamin, endophilin A1, and synaptojanin 1 (Wu Y. et al., 2014; Soykan et al., 2016; Watanabe et al., 2018; Chanaday et al., 2019). However, the precise time scale of the modes of SV recycling is highly debated (He and Wu, 2007; Soykan et al., 2017; Watanabe and Boucrot, 2017). It is generally accepted that an adaptor protein (such as adaptor protein 2, AP-2) is required for SV cargo detection and clathrin recruitment to initiate the most classic form of endocytosis. Meanwhile, clathrin-mediated endocytosis is considered the main mode of SV recycling (Soykan et al., 2016; Chanaday et al., 2019). From this point of view, the origin of synaptic vesicles from the cell membrane is SVE, in preparation for neurotransmission (Saheki and De Camilli, 2012). We will first outline the steps involved in this SVE process.

Many molecules and their families, such as endophilin, dynamin, synaptojanin1, and auxilin, including mutual interaction, are involved in the endocytic process to mediate membrane deformation, fission and clathrin uncoating. Generally, the clathrin-dependent endocytosis process can be divided into four steps (Mcmahon and Boucrot, 2011; Ramanan et al., 2011): (1) nucleation of clathrin-coated pits; (2) cargo capture; (3) curvature induction and membrane invagination or coat growth; (4) vesicle scission and clathrin uncoating. SVE begins by recruiting adaptor proteins to the cytoplasmic surface of the plasma membrane to the PtdsIns(4,5)P_2_ lipid-enriched region (Kelly et al., 2014). These adaptor proteins (such as AP-2, AP180, and epsin) regulate cargo sorting to ensure proper protein internalization with vesicles. Next, the membrane benders such as BAR domain proteins are recruited to the plasma membrane, where they mediate the invagination of vesicles (Frost et al., 2009; Trempe et al., 2009; Ramanan et al., 2011; Galic et al., 2012; Pechstein et al., 2015). The vesicles are generated from the plasma membrane, which is rich in PtdsIns(4,5)P_2_, and contains various inositolphosphates, which mediates the recycling of vesicles (Rohrbough and Broadie, 2005). Endophilin A1 could subsequently interact with dynamin and recruits synaptojanin 1 to the membrane interface (Schuske et al., 2003; Verstreken et al., 2003; Watanabe et al., 2018). Dynamin then constricts the neck and mediates clathrin-coated vesicles (CCVs) fission from the plasma membrane, through its GTPase activity (Marks et al., 2001; Daumke et al., 2014). When the CCVs are free, synaptojanin 1 exerts 5′-phosphatase activity to dephosphorylate PtdsIns(4,5)P_2_ to PtdsIns4P, which can be further dephosphorylated to PtdsIns (Chang-Ileto et al., 2011; Saheki and De Camilli, 2012). Dephosphorylation of PtdsIns(4,5)P_2_ causes the release of AP-2, which relies on PtdsIns(4,5)P_2_ for its vesicle binding, and allow auxilin to bind the CCVs through its PTEN-like and clathrin-binding domains (Yim et al., 2010; Saheki and De Camilli, 2012). Auxilin is a cofactor for HSC70, which simulates the removal of the clathrin coat through its ATPase activity (Morgan et al., 2001; Eisenberg and Greene, 2007; Yim et al., 2010; Saheki and De Camilli, 2012). Once the clathrin coat is fully removed, dopamine or other neurotransmitters can be packaged into the nascent vesicle, and quickly transported to synaptic vesicle pools in anticipation of the next neuronal stimulation (Ramanan et al., 2011; Saheki and De Camilli, 2012).

The SVE-dependent regeneration of synaptic vesicles is a highly regulated process and essential to restore neurotransmission. SVE is a unique system that is regulated by the phosphorylation-dephosphorylation-phosphorylation events of protein and lipids (Cousin and Robinson, 2001). These proteins include dynamin, synaptojanin1, amphiphysin 1 and 2, and epsin (Cousin and Robinson, 2001). Under the quiescent condition, SVE proteins are constitutively phosphorylated, thereby inhibiting their association to other proteins in the endocytic pathway (Cousin and Robinson, 2001). When neurons are stimulated, Ca^2+^ flows into the cell and activates Ca^2+^-dependent calcineurin activity, which rapidly dephosphorylates endocytic proteins, allowing them to interact and recruit to endocytic sites (Cousin and Robinson, 2001; Wu X.S. et al., 2014; Cottrell et al., 2016). SVE protein inactivation through rephosphorylation occurs on a much slower timescale in a stepwise process. This process has been shown to be mediated in part by Cdk5 and Minibrain kinase (Tan et al., 2003; Chen et al., 2014). However, other kinases may also participate in the proper time/space control of SVE in neural terminals (Pelkmans et al., 2005).

## Axon Degeneration and SVE Dysfunction in PD

Emerging evidences from human post-mortem studies, functional neuroimaging, genetic studies and neurotoxin models show that axon degeneration and SVE dysfunction may be the earliest feature of the disease (Lee et al., 2000; Serra et al., 2002; Dauer and Przedborski, 2003; Shin et al., 2008; Li L.H. et al., 2009; Qing et al., 2009; Cheng et al., 2010; Kagi et al., 2010; Schulz-Schaeffer, 2010; Peelaerts et al., 2015). Furthermore, the molecular mechanisms of degeneration of axons are distinct from those of neuron soma (Burke and O’malley, 2013). SVE dysfunction is one of presynaptic terminal degeneration mechanisms. But as SVE is not unique to dopaminergic neurons in the ventral midbrain, the specific vulnerability of these neurons to SVE deficiency in PD remains unclear. PD-linked *SYNJ1* R258Q mouse models revealed delays in SVE and marked changes in dopaminergic axon terminals in the dorsal striatum, highlighting a region-specific vulnerability of these neurons to synaptojanin 1 dysfunction (see later) (Cao et al., 2017). In *LRRK2* G2019S transgenic mice, SVE dysfunction including accumulation of CCVs and decreased synaptic densities were found specifically in dopaminergic pathway, resulting in axon degeneration and selective impairment of dopaminergic neurons (Pan et al., 2017). One possibility might be the sensitivity of dopaminergic neurons to the accumulation of cytosolic dopamine. Cytosolic dopamine is an oxidative stressor, suggesting that dopamine itself is a factor of particular vulnerability to numerous synaptic components (Chen et al., 2008). Hence, disruption of SVE in PD models may interrupt normal dopamine homeostasis by lowering dopamine loading into cycling SVs, resulting in increased cytosolic dopamine that is toxic. In a rat model, acute overexpression of human α-synuclein led to the specific degeneration of striatal terminals without nigral cell death (Busch et al., 2014; Eguchi et al., 2017; Medeiros et al., 2017). Moreover, dopamine is packaged into synaptic vesicles regenerated from SVE using a proton gradient created by vATPases located on the membrane surface of synaptic vesicles (Eiden et al., 2004). vATPase activity is dramatically inhibited by the clathrin coat and immediately restored once the coat was removed by auxilin (Farsi et al., 2018). Therefore, CCVs can be uncoated by means of a series of synaptic proteins including synaptojanin 1 and auxilin, and turned into SVs (Morgan et al., 2002). These newborn SVs will be loaded with other neurotransmitters such as dopamine, stored in the release vesicles pool, and/or fused with the plasma membrane to complete exocytosis (Morgan et al., 2002). The above evidence indicates that the dysfunction of SVE may cause dopamine to be incorrectly packed into vesicles, leading to increased cytosolic dopamine, ultimately contributing to dopaminergic neurodegeneration beginning at the axon terminals.

## PD Genes Involved in SVE at the Nerve Terminal

Parkinson’s disease is considered as a multifactorial disease caused by both genetic and environmental factors. Most of the PD patients are sporadic, but about 5% of them are caused by genetic mutations. More than 20 genes have been found to cause PD. Interestingly, some PD-associated genes, including *SNCA*, *LRRK2*, and *PRKN*, may also be potential regulators of SVE.

### α-Synuclein in SVE

α-Synuclein, encoded by the *SNCA* gene, is the major protein component of the Lewy bodies in the brain of PD patients. It is a small protein (14 kDa) consisting of three functional domains: a highly conserved N-terminal domain that can form an amphiphilic α-helix for lipid interactions, a central hydrophobic NAC structure domain, which is involved in the formation of β-sheet-rich amyloid fibrils, and the unfolded C-terminal tail, which is known to bind VAMP2/synaptic short fibers 2 (Davidson et al., 1998; Giasson et al., 2001). All PD-related mutations of SNCA, A30P, E46K, H50Q, G51D, and A53T are located in the amphiphilic region, suggesting the role of α-synuclein/lipid interactions in the pathogenesis of PD (Polymeropoulos et al., 1997; Kruger et al., 1998; Zarranz et al., 2004; Appel-Cresswell et al., 2013; Lesage et al., 2013; Proukakis et al., 2013).

α-Synuclein is mainly distributed in the presynaptic nerve terminals in the cerebral cortex, hippocampus and striatum. It mostly presents in the synaptic vesicle fraction (Lee et al., 2008). Coincidentally, the early pathological change detected during the progression of PD is the presynaptic degeneration of dopaminergic neurons in the striatum, long before the death of the cell bodies (Fahn, 2003; Sossi et al., 2004; Kurowska et al., 2016). It is now generally accepted that α-synuclein binds to synaptic vesicles. The evidence on its role in sensing and stabilizing the curvature of these tiny organelles is much more recent and still emerging. Regarding the function of α-synuclein in vesicle endocytosis, different views are emerging (Lautenschlager et al., 2017).

In the sucrose density gradient centrifugation experiment, wild-type α-synuclein, its mutant A30P α-synuclein, and the vesicle marker molecule synaptophysin were all present in the low-density component of synaptic proteins. α-Synuclein is believed to be a soluble protein located at the presynaptic end and is involved in the regulation of synaptic activity, plasticity, and transport of synaptic vesicle pools (Rizzoli and Betz, 2005; Lautenschlager et al., 2017, 2018). The pre-synaptic distribution of α-synuclein appears after synapse formation, indicating that α-synuclein has no decisive effect on the formation of synapses. However, when the normal expression of α-synuclein is suppressed, the number of vesicles in the vesicle recycling pool and vesicle reserve pool farther from the active region in the presynaptic tip is reduced (Rizzoli and Betz, 2005), but the number of easily released vesicles which are near or anchored in the active area is not affected (Murphy et al., 2000). In the electrophysiology experiments, undertaking long-term stimulation, the release of vesicles from α-synuclein knockout mice is less than that of wild-type mouse, indicating that in the absence of α-synuclein in the presynaptic terminals of neurons, the number of vesicles in the vesicle circulation region is not maintained and replenished in a timely manner (Barbiroli et al., 2002). But acute injection of human wild-type monomeric α-synuclein into lamprey synaptic terminals strongly stimulates the neurons, which can significantly reduce the rate of endocytosis, leading to CCVs accumulation (Busch et al., 2014) and synaptic fidelity in the calyx of held (Eguchi et al., 2017). Taken together, this indicates that maintenance of normal α-synuclein expression levels is necessary to ensure an appropriate number of synaptic vesicles, in the presynaptic terminals.

Except for the monomeric α-synuclein mentioned above, different α-synuclein conformations or strains may also exert distinct effects on SVE. For example, α-synuclein dimers lead to the formation of unique clathrin-coated pits (Medeiros et al., 2017). Multimers of α-synuclein may affect the early stages of CCVs formation and fission, while monomeric α-synuclein may affect the final step of clathrin-uncoating (Wang et al., 2014). Further studies found that α-synuclein is an important regulator of presynaptic terminal size and organizer of different synapses vesicle pools by specifically regulating between the synaptic vesicles and the plasma membrane (Vargas et al., 2017). In recent years, studies on the relationship between α-synuclein and proteins in the SNARE complex indicate that it plays a role in regulating the release of synaptic vesicles (Choi et al., 2013). The interaction of α-synuclein and SNARE protein complex regulates the release of neurotransmitters from presynaptic vesicles (Darios et al., 2010; Burre et al., 2014). These studies together suggest that α-synuclein plays an important role in regulating the recycling of synaptic vesicles by endocytosis.

### *LRRK2* in SVE

In 2002, scientists identified a mutation in *LRRK2* (*PARK8*), an important pathogenic gene on chromosome 18 in a family of Japanese patients with hereditary PD (Funayama et al., 2002). Two years later, two groups reported that two LRRK2 mutations also existed in two unrelated families of PD patients in Europe (Paisan-Ruiz et al., 2004; Zimprich et al., 2004). Up to now, several mutations of LRRK2, such as R1396G, R1441C, Y1654C, Y1699C, 11122V, I2020T, and G2019S have been reported to increase the incidence of PD. Transgenic mice expressing the R1441C mutant LRRK2 can partially replicate the phenotype of PD, such as decreased dopamine levels and abnormalities in the nigrostriatal projection (Li Y. et al., 2009). The phenotypic study of LRRK2 G2019S mutant transgenic mice suggests that although there is no significant loss of dopaminergic neurons in the substantia nigra, the dopamine content and dopamine release in the striatum and intake levels were decreased significantly (Li et al., 2010). Overexpression of mutation LRRK2 G2019S in mouse brain induced PD-like pathology (Xiong et al., 2017). Introducing G2019S mutant LRRK2 in stem cells derived from patients with PD can lead to degenerative phenotype. These results indicate that mutant *LRRK2* can directly cause PD-related changes (Liu et al., 2012).

Although the cause of PD induced by mutations in the LRRK2 gene is not clear, there is some evidence to support the hypothesis that LRRK2 protein, like α-synuclein, can affect the dopamine system by regulating the recycling of presynaptic vesicles. LRRK2 protein exists in synaptic components rich in vesicles and membrane structures in the rat brain, and is distributed by dots in synapses (Biskup et al., 2006). It can be speculated that it may be related to the cell membrane and synaptic vesicle structure. Chemical inhibition of LRRK2 has been shown to delay endocytosis, indicating normal serine/threonine kinase activity of LRRK2 is essential for proper SVE (Arranz et al., 2015). In addition, *LRRK2* mutant mouse showed an accumulation of CCVs and reduced synaptic vesicle density in dopaminergic neuronal terminals (Xiong et al., 2018).

Due to the lack of a distinct transmembrane region, LRRK2 may loosely bind to synaptic vesicles by interacting with membrane and presynaptic proteins. Recent studies have identified some proteins that are specifically involved in SVE regulation, and are interacting partners and/or substrates of LRRK2, such as dynamin, Rab5b (one of Rab proteins), endophillin A1, and synaptojanin 1. The major function of dynamin is mediating CCVs to fusion from the plasma membrane. LRRK2 has been reported to interact with dynamin, and regulate its GTPase activity (Stafa et al., 2014). LRRK2 achieves its effect on synaptic vesicle circulation by interacting with vesicle endocytosis-related proteins Rab5b and endophillin A1 (Shin et al., 2008). Moreover, LRRK2 can phosphorylate endophillin A1 at positions T73 and S75 in its BAR domain, and LRRK2-mediated phosphorylation of S75 endophillin A1 plays a key role in mediating the function of endophillin A1 in synapses (Matta et al., 2012; Ambroso et al., 2014). Recent synaptic proteomic analysis of the *LRRK2* mutant *Drosophila* model has also identified synaptojanin 1, which is essential for synaptic vesicle recycling, as a substrate of LRRK2 kinase (Islam et al., 2016). LRRK2 phosphorylates synaptojanin 1 at positions T1131 and T1205 located in its PPR domain, resulting in defective synaptojanin 1-endophilin A1 interaction (Islam et al., 2016; Pan et al., 2017). To sum up, LRRK2 kinase activity can regulate many steps of SVE, and the pathogenic mutation of LRRK2 may aggravate the deficient of SVE, leading to downstream toxic effects and thus degeneration of dopaminergic neurons.

### Parkin in SVE

Parkin mutation was found in patients with autosomal recessive PD (Kitada et al., 1998). Parkin, encoded by *PRKN* (also known as PARK2), is a component protein in the ubiquitin ligase complex. Its mutation causes the malfunction of ubiquitin ligase, which causes the deposit of its substrate (such as α-synuclein, etc.) in neurons (Shimura et al., 2001). Parkin is widely distributed in the brain. Unlike α-synuclein and LRRK2, it is evenly distributed in the cytoplasm of neurons without enriching the presynaptic terminals (Shimura et al., 1999). *PRNK* knockout mouse shows abnormalities in neurotransmission (Periquet et al., 2005). As an E3 ubiquitin ligase, Parkin has been shown to ubiquitinate endophilin A1, whose major binding partners are dynamin and synaptojanin 1 (Cao et al., 2014). It has been reported that the ubiquitination of Parkin is responsible for regulating the expression level of endophilin A1. It can also regulate protein’s ability to recruit and bind its interaction partners at the plasma membrane and CCV interface (Trempe et al., 2009; Cao et al., 2014). These data suggest that Parkin E3 ubiquitin ligase function may be an important regulator of synaptic vesicle recycling, and *PRKN* mutations that cause loss of E3 ligase function may negatively affect the process of SVE.

### *PINK1* in SVE

The mutations of *PINK1* (*PARK6*) and *DJ-1* (*PARK7*) genes cause recessive familial PD (Valente et al., 2004). It is believed that these mutations cause fewer cases of recessive familial hereditary PD than Parkin mutations. The molecular mechanisms underlying PINK1 and DJ-1 mutation remain unclear. However, both of them are closely related to synaptic vesicle activity. In *Drosophila* neurons, PINK1 dysfunction does not change the basic level of synaptic vesicle release, but vesicles in the vesicle pool cannot be efficiently transported to the active zone to be released when the synapse is rapidly and strongly stimulated, causing the synaptic release to become weaker compared to wild type after long-term stimulation (Morais et al., 2009). PINK1 mutants exhibited reduced levels of other proteins involved in synaptic transmission, and the synaptic vesicles with PINK1 mutants had broken membranes (Doktor et al., 2018). The lack of PINK1 leads to increased hippocampal excitatory transmission and release of neurotransmitters, which may lead to cognitive impairments in PD (Feligioni et al., 2016). PINK1 mutants have disrupted synaptic mitophagy, which is crucial in maintaining the health of the pre-synaptic terminal (Vidyadhara et al., 2019). PINK1 and parkin have also been reported to associate with lipid rafts, implying that functional alteration of lipid rafts by these proteins may regulate lipid rafts-dependent endocytosis (Silvestri et al., 2005; Cha et al., 2015).

### *DJ-1* in SVE

DJ-1 has been reported to be closely linked to the plasma membrane and interact with synaptophysin and Rab3A, the synaptic vesicle proteins (Usami et al., 2011). DJ-1 deficiency impairs synaptic vesicle endocytosis and re-availability in synapses, without structural alterations (Kyung et al., 2018). DJ-1 mutants (M26I, E64D, and L166P) were unable to rescue the endocytosis defects of synaptic vesicles, while the expression of wild-type DJ-1 completely restored the endocytic function of DJ-1 KO neurons (Kyung et al., 2018). These results indicate that DJ-1 is essential for synaptic vesicle endocytosis and reavailability, and impairment of this function by PD-associated DJ-1 mutants may be related to the pathogenesis of PD.

In shorts, more and more evidence has been found to suggest that PD-related genes including *SNCA*, *LRRK2*, *PRKN*, *PINK1*, and *DJ-1* are involved in synaptic vesicle exocytosis, endocytosis, and recycling. The role of α-synuclein in the process of vesicle activity has been most extensively studied. There are many reports on the role of LRRK2 in the process of SVE, but the underlying mechanisms remain unclear. The study of Parkin, PINK1 and DJ-1 genes is relatively rare.

### Mutations of SVE Genes in PD

Recently, several SVE-related genes have been discovered to be involved in PD, further supporting the importance of SVE defects in the pathogenesis of PD.

### Mutations of *DNAJC6* in PD

*DNAJC6* encodes auxilin, which is an enzyme of putative tyrosine-protein phosphatase. Auxilin is a cofactor for hsc70 and its J domain is responsible for recruitment of the ATPase to stimulate clathrin-coat removal (Saheki and De Camilli, 2012; Nguyen and Krainc, 2018). Mutations in *DNAJC6* were initially described in atypical Parkinsonism patients (Edvardson et al., 2012). Homozygous mapping of two patients with juvenile PD showed that a harmful splice site mutation in *DNAJC6*, c.801-2 A > G, resulted in a significant decrease in mRNA levels (Edvardson et al., 2012). Another study found that one patient had a *DNAJC6* homozygous truncated mutation Q734X, which caused almost 20% loss of the C-terminus, including its functional J domain that is responsible for binding to HSC70 (Koroglu et al., 2013). Recent studies have reported other *DNAJC6* mutations, such as R927G and T741T, are associated with early onset PD cases (Olgiati et al., 2016). These disease-related mutations link *DNAJC6* to the pathogenesis of PD. These mutations result in reduced auxin expression and are expected to reduce their overall function (Olgiati et al., 2016).

### Mutations of *SYNJ1* in PD

Synaptojanin 1 is an inositol phosphatase enriched at the presynaptic terminals. Synaptojanin 1 is a 145-kDa protein that contains three functional domains: a SAC1-like domain that hydrolyzes inositol monophosphates such as PI3P and PI4P; a 5’-phosphatase domain that hydrolyzes PI (4, 5)P_2_ to produce PI4P; and a highly variable C-terminal proline-rich domain that can be recruited by endophilin A1 during SVE. All three domains are involved in regulating the SVE at various stages of synaptic activity (Mani et al., 2007). The Sac1 domain homologous to yeast SacIp mainly dephosphorylates phosphatidylinositol monophosphate present in organelle membranes (including the membranes of the Golgi apparatus and endosomes) to recruit proteins required for membrane transport. The 5-phosphatase domain hydrolyzes the phosphate group at the D-5 position of phosphatidylinositol diphosphate or triphosphate located on the plasma membrane to activate endocytosis or cytoskeletal reorganization, and other pathways (Mcintire et al., 2014). SYNJ1 is necessary for normal SVE and membrane passage, and controls the number and release time of vesicles in the synapses of sensory neurons (Drouet and Lesage, 2014). Cytoplasmic SYNJ1 protein was directed to endocytic sites thanks to protein-protein interactions through its C-terminal PRD domain with Src homology 3 (SH3) domain-containing proteins. The C-terminal region to SYNJ1 was shown to interact with the SH3 domains of a variety of proteins implicated in synaptic vesicle recycling and trafficking, subcellular targeting, and signaling such as endophilin, amphiphysins, or intersectin. These domains play their role in SVE by interacting with their phosphohydrolases and endocytosis- related proteins.

The association of R258Q and R459P mutations in *SYNJ1* with juvenile or early onset PD was recently reported in several independent studies (Krebs et al., 2013; Quadri et al., 2013). A homozygous R258Q mutation of *SYNJ1* caused early onset PD in an Italian family. Knock-in mice carrying the homozygous R258Q disease allele, which abolishes the SAC1 activity required for hydrolyzing PI3P and PI4P, recapitulated parkinsonian symptoms and exhibited defective clathrin uncoating of SVs and dystrophic changes in the nigrostriatal terminals (Krebs et al., 2013; Cao et al., 2017). The mutation of R459P is the same as that of R258, and the mutation sites are all in the Sac-like domain. If these sites are mutated, they will affect the function of the Sac1 domain, and the function of SYNJ1 will also be problematic. *Synj1* heterozygous deletion (*Synj1*^±^), which is associated with an impaired 5′-phosphatase activity, also leads to Parkinson’s disease (PD)-like pathologies in mice (Pan et al., 2020). A homozygous mutation of R459P in *SYNJ1* had been identified in an Indian family with autosomal recessive juvenile Parkinsonism (Olgiati et al., 2014; Kirola et al., 2016). These mutations are located in the Sac1 domain of synaptojanin 1 and impair its mulitphosphatase activity (Krebs et al., 2013; Quadri et al., 2013; Olgiati et al., 2014). Interestingly, the insufficient haploid function of synaptojanin 1 caused delayed SVE of dopaminergic neurons in the midbrain of mice, but not cortical neurons, suggesting that the loss of synaptojanin 1 function may specifically affect vulnerable dopaminergic neurons in the pathogenesis of PD. Moreover, through genetic engineering techniques, these mutant mouse models have PD phenotypes, such as age-dependent motor function abnormalities as well as α-synuclein accumulation, impaired autophagy and dopaminergic terminal degeneration (Cao et al., 2017; Pan et al., 2020). These results show that *SYNJ1* plays an important role in SVE, and its variants play a more critical role in the pathogenesis of PD. Further research is needed on the role of *SYNJ1* in the development of PD.

### Synaptic Gene *SH3GL2* Mutation in PD

Recently, a large-scale GWAS meta-analysis reported that *SH3GL2* (encode endophilin A1) was identified as a PD risk factor (Chang et al., 2017). Endophilin A1 is involved in SVE regulation. Moreover, research in 2018 confirmed that not only the variant of *SH3GL2*, but also changes in the binding domain of its microRNA can cause the onset of PD (Ghanbari et al., 2016). In addition, earlier studies have confirmed that *SH3GL2* plays a key role in the normal function of CNS. Its dysfunction is involved in the occurrence and development of PD disease (Shi et al., 2009). However, even though the physiological function of endorphin A1 in neural terminals is well understood, its specific molecular mechanism in the pathogenesis of PD remains unclear.

## Animal Models Based on SVE Genes Demonstrate Distinct PD Phenotypes

Mouse models knockout of *DNAJC6*, *SYNJ1*, and *SH3GL2* all showed endocytosis defects at the synapses, highlighting the importance of proper SVE control in maintaining axonal terminal integrity (Kim et al., 2002; Yim et al., 2010; Milosevic et al., 2011). Previous reports have shown that presynaptic compartments in auxilin knockout mice display SVE-deficient features, including reduced synaptic vesicle density, increased CCVs, and membraneless clathrin cages (Yim et al., 2010). However, subsequent studies found that genetic ablation of the auxilin homolog of GAK(Cyclin-G-associated kinase) and *DNAJC6* can lead to lethality in embryonic mice, while overexpression of the C-terminal fragment and J domain of GAK binding to clathrin can rescue the phenotype of the mice (Beilina et al., 2014; Nalls et al., 2014; Park et al., 2015). In cell models, clathrin was additionally coat to vesicle by GAK and auxilin. Therefore, these evidences suggest that auxilin induced dysfunction in PD could be potentially rescued by overexpression of GAK. In addition, the lack of auxilin in *Drosophila* causes age-related motor dysfunction and accelerates α-synuclein-mediated dopaminergic neuron loss (Song et al., 2017). These results indicate that the dopaminergic neurons are more sensitive to the loss of auxilin function. Consistent with previous reports, synapses in *SYNJ1* R258Q knock-in mice show severe endocytosis defects and more CCVs and other endocytosis intermediates (Cao et al., 2017). Moreover, dystrophic neural terminals were observed in the dorsal striatum of these mice, which is the main site of substantia nigra compact dopaminergic neuron projection in the brain. In addition, auxilin and parkin levels are reported to be elevated in *SYNJ1* mutant mice (Cao et al., 2017). *Synj1* heterozygous deletion (*Synj1*^±^), which is associated with an impaired 5’-phosphatase activity, also leads to Parkinson’s disease (PD)-like pathologies in mice (Pan et al., 2020). Endophilin A triple knockout (TKO) mice were fatal shortly after birth. Mice with endophilin A deficiency exhibit age-dependent dyskinesia and progressive ataxia, reminiscent of neurodegeneration in endophilin A1 and endophilin A2 double deletion (DKO) mice phenotype (Tanasic et al., 2011; Murdoch et al., 2016). Mouse models of the endophilin A1 gene knockout also show elevated levels of parkin and CCV accumulation, highlighting the potential functional connection between these SVE proteins and parkin (Kjaerulff et al., 2011). These transgenic animal models once again demonstrate that SVE-related genes are involved in the onset and development of PD.

## Other Pathways Involved in Regulation by SVE

**FIGURE 2:**
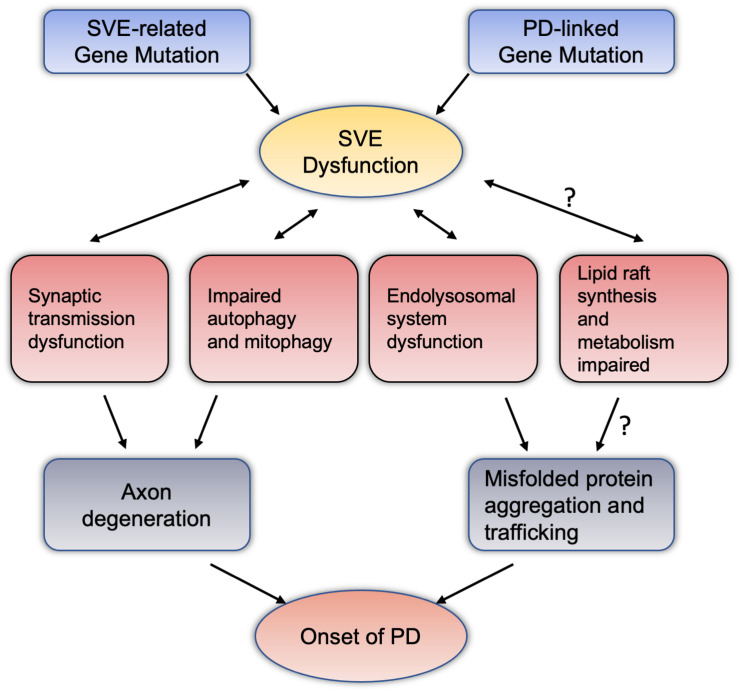
Synaptic vesicle endocytosis dysfunction potentially mediate dopaminergic neurodegeneration through intersections with various pathway. Mutations in the PD-related genes or SVE-related genes reviewed in this article may cause damage to the endocytosis of vesicles at the terminal axons of neurons. SVE may interact with synaptic transmission, autophagy and mitochondrial autophagy, and the endolysosomal system has the effect of this two-way condition. These pathways can lead to the accumulation and spread of misfolded proteins with axon degeneration or neurotoxicity, and ultimately lead to the pathogenesis of PD. Among them, regarding the relationship between SVE and lipid raft synthesis and metabolism, the mechanism of abnormal lipid raft metabolism leading to the pathogenesis of PD deserves more research and attention. PD, Parkinson’s disease; SVE, synaptic vesicle endocytosis.

We summarized PD-related genes involved in the regulation of SVE, and SVE-related gene mutations are involved in the pathogenesis of PD. It is believed that SVE is essential to maintain the homeostasis and health of the presynaptic membrane. If SVE dysfunction, it may lead to axon degeneration, leading to the onset of PD. In fact, in the pathogenesis of PD, the accumulation and spread of misfolded proteins, and Lewy Body formation, are the major mechanisms (Mahul-Mellier et al., 2020). The function of SVE is also related to the accumulation and spread of misfolded proteins, such as α-synuclein. Among them, SYNJ1, the above-mentioned important endocytic protein, its heterozygotes may promote the aggregation and high phosphorylation of α-synuclein (Pan et al., 2020). The dysfunction of SVE may be to only the dysfunction of endolysosomal system, which leads to the spread of the misfolded protein (Vidyadhara et al., 2019). Furthermore, SVE is an important physiological mechanism involved in the balance and transformation of lipids, phosphatidylinositol in synapses, axons, and even the soma. SVE also involves organelles and other physiological functions, such as autophagy and mitophagy. If SVE is dysfunctional, autophagy and mitophagy would also have abnormal performance, which will inevitably affect the normal functions of synapses and axons (Pan et al., 2020). Besides the regulation of SVE at the synapse, several endocytic genes have also been identified as critical modulators of synaptic autophagy, a pathway for the maintenance of synaptic protein homeostasis and turnover via the lysosome following neurotransmission (Soukup et al., 2018). Likewise, mitochondria, an important organelle, may be due to the decrease of ATP and the increase of reactive oxygen species due to SVE dysfunction, which further leads to the onset of PD (Nguyen et al., 2019). In the study of lipids, studies have shown that the possibility of lipid rafts is also involved in the aggregation and positioning of α-synuclein (Brummel et al., 2017). Dysregulation of lipid rafts-dependent endocytosis may caused cell-to-cell transmission of α-synuclein was facilitated (Cha et al., 2015). SVE-related proteins, such as SYNJ1, are involved in the metabolic balance of phosphatidylinositol, whether it will affect lipid rafts, whether they will participate in the aggregation of asyn and the spread of pathological asyn, which are worthy of further research and discussion. Based on these evidences and speculations, we summarized the pathways and physiopathological functions that SVE may be involved in the pathogenesis of PD (see Figure 2).

## Conclusion and Perspectives

The pathogenesis of PD remains unclear, which presents a major obstacle to the development of neuroprotective therapies. SVE dysfunction is involved in presynaptic nerve terminal degeneration. Emerging evidence suggests that the dysfunction of SVE is tightly related to the onset of PD. Several PD-related genes were found to play a role in regulating SVE and synaptic function, while at the same time, mutations in key genes of SVE play a role in the pathogenesis of PD (see Figure 1 and Table 1). SVE dysfunction involves the degeneration of presynaptic nerve terminals, autophagy and mitophagy disorder, and through a variety of ways and means, participates in the abnormal aggregation and transport of neurotoxic misfolded proteins. Participating in the above changes, these mechanisms can indicate that SVE dysfunction may be involved in the pathogenesis of PD (see Figure 2). Although existing evidence suggests that SVE is regulated by a set of overlapping genes, how these are converted to regulate different pathways remains to be elucidated. More studies are needed to illustrate the role of SVE in regulating synaptic function and PD pathology. It should be noted that the mammalian CNS neurotransmission systems are highly complex in time and space, making the illustration of its role in PD more difficult. Further research is needed to address these important issues. An in-depth understanding of the interaction between SVE and multiple signaling pathways or key mechanisms may be the most appropriate target for early diagnosis and provide possible new targets for therapeutic intervention of PD.

**TABLE 1:** Summary of PD-linked and SVE-associated genes information in this article.

Gene	Location	Protein	Function	Mutation	Animal model involved in the pathogenesis of PD
*SNCA*	(Human) Chromosome 4, 4q22.1 (Mouse) Chromosome 6, 6 B3| 6 29.15 cM	α-Synuclein	The major protein component of the Lewy body in the brain of patients with PD.	A30P, E46K, H50Q, G51D, and A53T	Mice expressing mutant α-synuclein, or overexpression of wild-type α-synuclein, SNCA knockout model, acute injection of virus encoding wild-type or mutant α-synuclein, or α-synuclein pre-formed fibrils
*LRRK2*	(Human) Chromosome 12, 12q12 (Mouse) Chromosome 15, 15| 15 E3	Leucine-rich repeat kinase 2	LRRK2 is a kinase that is located largely in the cytoplasm but also associates with the mitochondrial outer membrane. LRRK2 interacts with the C-terminal R2 ring finger domain of Parkin	R1396G, R1441C, Y1654C, Y1699C, I1122V, I2020T, and G2019S.	Mice with G2019S R1441C mutant. Chemical inhibition of LRRK2. LRRK2 knockout mice
*PRKN*	(Human) Chromosome 6, 6q26 (Mouse) Chromosome 17, 17A1| 17 7.8 cM	Parkin	Parkin is a 465-residue E3 ubiquitin ligase that plays a critical role in ubiquitination- the process whereby molecules are covalently labeled with ubiquitin and directed toward degradation in proteasomes or lysosomes.	R256C, R275W, G328E, A398T, and T415N; heterozygous mutation	Parkin knockout mice
*PIKN1*	(Human) Chromosome 1, 1p36.12 (Mouse) Chromosome 4, 4| 4 D3	PTEN-induced kinase 1	PINK1 is a mitochondrial serine/threonine-protein kinase, which is thought to protect cells from stress-induced mitochondrial dysfunction. PINK1 activity causes the parkin protein to bind to depolarized mitochondria to induce autophagy of those mitochondria. PINK1 is processed by healthy mitochondria and released to trigger neuron differentiation	R246X, H271Q, E417G, L347P, and Q239X/R492X heterozygous *PINK*1 mutations	*PINK1* knockout mice, PINK1-deficient mouse and lack of PINK1 in *Drosophila*
*DJ-1*	(Human) Chromosome 1, 1p36.23 (Mouse) Chromosome 4, 4| 4 E2	human protein deglycase DJ-1	DJ-1 protects neurons against oxidative stress and cell death. DJ-1 acts as a positive regulator of androgen receptor-dependent transcription.	M26I, E64D, and L166P	*DJ-1* knockout mice
*DNAJC6*	(Human) Chromosome 1, 1p31.3 (Mouse) Chromosome 4, 4| 4 C6	Auxilin	Auxilin regulates molecular chaperone activity by stimulating ATPase activity. Auxilin is a putative tyrosine-protein phosphatase.	Q734X, R927G, and T741T	*DNAJC6* knockout mouse Auxilin knockout *Drosophila*
*SYNJ1*	(Human) Chromosome 21, 21q22.2 (Mouse) Chromosome 16, 16| 16 52.18 cM	Synaptojanin 1	Synaptojanin 1 is a protein involved in vesicle uncoating in neurons. This is an important regulatory lipid phosphatase.	R258Q and R459P Homozygous R258Q mutation	*SYNJ1* R258Q knock-in mice *SYNJ1* knockout *SYNJ1* heterozygote and triploid
*SH3GL2*	(Human) Chromosome 9, 9p22.2 (Mouse) Chromosome 4, 4 C4| 4 40.23 cM	Endophilin A1	Endophilin A1 is a member of the BAR protein family involved in membrane deformation, and its SH3 domain in the carboxyl-terminal is capable of recruiting dynamin and synaptojanin 1	F10E, A66D, and A63S/A66S/M70Q (or called “SSQ”) heterozygous mutation	EndoA triple knockout (TKO) mice Endophilin knockout mice heterozygous mutation of endophilin in *Drosophila*

## Glossary

**Amphipathic helix:**α-helical protein structure segregating hydrophobic and polar residues.

**Adaptor protein:** Here specifically refers to clathrin adaptor protein, also known as adaptation protein, which is a vesicle transport adaptor protein related to clathrin.

**BAR domains:** In molecular biology, BAR domains are highly conserved protein dimerization domains that occur in many proteins involved in membrane dynamics in a cell. The BAR domain is banana-shaped and binds to membrane via its concave face. It is capable of sensing membrane curvature by binding preferentially to curved membranes.

**Bulk endocytosis:** membrane uptake by large membrane invagination.

**Clathrin-mediated endocytosis**: one of endocytosis, which is characterized by a coating of vesicles with the clathrin-triskelion scaffold.

**E3 ubiquitin ligase**: A ubiquitin ligase is a protein that recruits an E2 ubiquitin-conjugating enzyme that has been loaded with ubiquitin, recognizes a protein substrate, and assists or directly catalyzes the transfer of ubiquitin from the E2 to the protein substrate.

**Endocytosis:** uptake of molecules from the extracellular space into the cell by forming cell invaginations. In the case of endocytosis at the synaptic terminal, the endocytosis is deemed to retrieve excess membrane rather than molecules.

**Endosomal-lysosomal system:** The endosomal-lysosomal system is made up of a set of intracellular membranous compartments that dynamically interconvert, which is comprised of early endosomes, recycling endosomes, late endosomes, and the lysosome.

**GAK: Cyclin-G-associated kinase**, the ubiquitously expressed J-domain protein, is essential for the chaperoning and uncoating of clathrin that is mediated by Hsc70.

**Kiss-and-run:** mechanism of exocytosis, where the synaptic vesicle opens only a small fusion pore. This pore reseals easily afterward and so the vesicle is kept intact. This in contrast to the full fusion event, where the synaptic vesicle collapses and both, vesicle and presynaptic membrane fuse together.

**LRRK2:** leucine-rich repeat kinase 2; enzymatic protein encoded by the PARK8 gene.

**Rab proteins:** proteins that present manifold involvement in membrane trafficking, including vesicle formation, transport and fusion.

## Author Contributions

LZ contributed to article writing and graphic design while YT helped with figure construction and tabulation, and ZZ provided fund support and manuscript proof. All authors read and approved the final manuscript.

## Conflict of Interest

The authors declare that the research was conducted in the absence of any commercial or financial relationships that could be construed as a potential conflict of interest.
